# Differential Response of Bacterial Microdiversity to Simulated Global Change

**DOI:** 10.1128/aem.02429-21

**Published:** 2022-03-22

**Authors:** N. C. Scales, A. B. Chase, S. S. Finks, A. A. Malik, C. Weihe, S. D. Allison, A. C. Martiny, J. B. H. Martiny

**Affiliations:** a Department of Ecology and Evolutionary Biology, University of California, Irvine, California, USA; b Center for Marine Biotechnology and Biomedicine, Scripps Institution of Oceanography, University of California San Diego, La Jolla, California, USA; c School of Biological Sciences, University of Aberdeen, Aberdeen, United Kingdom; d Department of Earth System Science, University of California, Irvine, California, USA; Georgia Institute of Technology

**Keywords:** bacteria, global change, microdiversity

## Abstract

Global change experiments often observe shifts in bacterial community composition based on 16S rRNA gene sequences. However, this genetic region can mask a large amount of genetic and phenotypic variation among bacterial strains sharing even identical 16S regions. As such, it remains largely unknown whether variation at the sub-16S level, sometimes termed microdiversity, responds to environmental perturbations and whether such changes are relevant to ecosystem processes. Here, we investigated microdiversity within *Curtobacterium*, the dominant bacterium found in the leaf litter layer of soil, to simulated drought and nitrogen addition in a field experiment. We first developed and validated *Curtobacterium*-specific primers of the *groEL* gene to assess microdiversity within this lineage. We then tracked the response of this microdiversity to simulated global change in two adjacent plant communities, grassland and coastal sage scrub (CSS). *Curtobacterium* microdiversity responded to drought but not nitrogen addition, indicating variation within the genus of drought tolerance but not nitrogen response. Further, the response of microdiversity to drought depended on the ecosystem, suggesting that litter substrate selects for a distinct composition of microdiversity that is constrained in its response, perhaps related to tradeoffs in resource acquisition traits. Supporting this interpretation, a metagenomic analysis revealed that the composition of *Curtobacterium*-encoded carbohydrate-active enzymes (CAZymes) varied distinctly across the two ecosystems. Identifying the degree to which relevant traits are phylogenetically conserved may help to predict when the aggregated response of a 16S-defined taxon masks differential responses of finer-scale bacterial diversity to global change.

**IMPORTANCE** Microbial communities play an integral role in global biogeochemical cycling, but our understanding of how global change will affect microbial community structure and functioning remains limited. Microbiome analyses typically aggregate large amounts of genetic diversity which may obscure finer variation in traits. This study found that fine-scale diversity (or microdiversity) within the bacterial genus *Curtobacterium* was affected by simulated global changes. However, the degree to which this was true depended on the type of global change, as the composition of *Curtobacterium* microdiversity was affected by drought, but not by nitrogen addition. Further, these changes were associated with variation in carbon degradation traits. Future work might improve predictions of microbial community responses to global change by considering microdiversity.

## INTRODUCTION

Microbial responses to global change experiments are often measured by observing shifts in the composition of operational taxonomic units (OTUs) based on 16S rRNA gene sequences (1–4). However, a high degree of genetic variation exists within such OTUs, variation here referred to as microdiversity (5–8). It remains unclear whether this variation among microdiverse lineages contributes to differential responses to environmental factors and whether such trait variation manifests at the functional level. For instance, microbiomes with disparate OTU composition can encode similar functions, suggesting few trait differences among 16S-defined taxa (9–11). On the other hand, taxa with identical 16S rRNA gene sequences can vary in ecologically important traits (12–16).

The importance of microdiversity for global change responses will likely depend on the traits involved (17). If an environmental perturbation selects for traits that are phylogenetically conserved at broader taxonomic levels, then the OTU designations would capture the consistent responses of the underlying microdiversity. Conversely, if a change selects on traits that vary at finer taxonomic levels, then 16S-level resolution will mask differential responses within the OTU. Therefore, identifying which bacterial traits are responsible for responses to environmental change and their degree of phylogenetic conservation will determine the level of genetic resolution needed to accurately assess compositional responses (18–20).

The Loma Ridge Global Change Experiment (LRGCE) in southern California simulates anthropogenically driven environmental changes expected in this region by experimentally manipulating rainfall and nitrogen in two adjacent plant communities: grassland and coastal sage scrub (CSS). Drought is projected to increase in intensity and frequency (21), while soil nitrogen deposition has steadily increased for decades due to human activities (22). Previous work at this site has shown that drought and nitrogen addition alter both bacterial and fungal community composition in the leaf litter (23, 24). However, these studies focused on the compositional responses at the community level, as assessed by 16S amplicon sequencing. To investigate whether bacterial microdiversity also responded to global change treatments, we revisited this site and focused on the response of one of the most abundant taxa.

*Curtobacterium* is a genus of Gram-positive, putative aerobic *Actinobacteria* highly abundant in leaf litter, the topmost layer of soil (25). Though originally known as a plant pathogen (26), *Curtobacterium* appears to be a major litter decomposer in southern Californian ecosystems and responds to both global change treatments at the LRGCE (6). Within the genus, the 16S region is highly conserved across this genus such that only four *Curtobacterium* OTUs are delineated at 100% sequence similarity of the hypervariable V4 region of the 16S region. These four OTUs comprise a high degree of genomic diversity, sharing <80% average nucleotide identity and representing at least nine distinct subclades based on genotypic and phenotypic characteristics (27). Using 16S amplicon sequencing, we found that the abundance of *Curtobacterium* (relative to all other bacterial taxa) increased in response to drought while exhibiting variable responses to nitrogen addition in the grassland and CSS ecosystems (28). Only two 100% OTUs (or ESVs, exact nucleotide variants) were detected, and one of these made up 98.9% of all the *Curtobacterium* sequences. However, using metagenomic sequencing, we also found that at least six subclades within the genus coexist at the field site (27). Thus, a 16S-level analysis cannot resolve within-*Curtobacterium* variation in the responses to the global change treatments.

To investigate the response of *Curtobacterium* microdiversity to simulated global change, we first needed to develop a new genetic marker to delineate and track the finer-scale genetic diversity within the genus. We created *Curtobacterium*-specific primers of the *groEL* gene, which after *in silico* analyses is expected to capture a higher degree of genetic variation than the 16S region. Thus, our first goal was to evaluate the *groEL* gene as a marker to capture *Curtobacterium* microdiversity and to distinguish between subclades defined by multiple marker genes. To assess the validity of this method, we compared the relative abundances of subclades derived from our *groEL* approach to those previously observed using shotgun metagenomes (6).

After initial validation of the *groEL* marker, we then tested two hypotheses. Based on the high degree of genomic variation within *Curtobacterium*, we first hypothesized that the previously observed response of *Curtobacterium* to simulated global changes masks disparate responses of its microdiversity. For instance, although *Curtobacterium* increased in relative abundance in response to drought overall, this aggregate response may obscure that some subclades responded differentially, with some being more drought tolerant than others. Moreover, we suspected that the response of *Curtobacterium* microdiversity to specific global change treatments will differ depending on the depth of conservation of the traits involved. In particular, drought responses of soil bacteria generally appear to be more deeply conserved than the 16S OTU level (20), whereas nitrogen assimilation is often highly variable; that is, it is shallowly conserved at the sub-OTU level (29, 30). In this case, *Curtobacterium* microdiversity would be altered by the nitrogen treatment, but not the drought treatment.

Second, we hypothesized that the response of *Curtobacterium* microdiversity to global change depends on other traits within the genus, especially those related to resource utilization strategies (31). Therefore, we expected that the microdiversity response to global change would depend on the ecosystem (grassland or CSS) because of differences in the leaf litter substrate. While both ecosystems experience similar abiotic conditions due to their close proximity, their leaf litter differs in polysaccharide content and selects for distinct bacterial communities (28). Genes encoding the breakdown of leaf litter, the carbohydrate-active enzymes (CAZymes), vary in their distribution and diversity among *Curtobacterium* subclades and are correlated with differential degradation rates of cellulose and xylan, two abundant carbohydrates in leaf litter (6). We thus further explored the role of CAZymes in this hypothesis by comparing the CAZyme diversity of fully sequenced *Curtobacterium* genomes to the response of *Curtobacterium* CAZymes from shotgun metagenomic libraries at the LRGCE.

## RESULTS

### Comparison of *Curtobacterium* microdiversity by two methods.

The *groEL* region captured a high degree of *Curtobacterium* microdiversity (12,228 exact sequence variants, including 6,184 singletons) while also providing a useful taxonomic marker of subclade diversity. For comparison, 16S sequencing of the same samples detected only two *Curtobacterium* exact sequence variants (ESVs). We also attempted to assess the microdiversity response to the LRGCE treatments using the 16S data. While the first ESV was found in all samples, sometimes as high as 30% relative abundance, the second ESV was found only in four samples, making any further 16S microdiversity analysis unfruitful.

**FIG 1:**
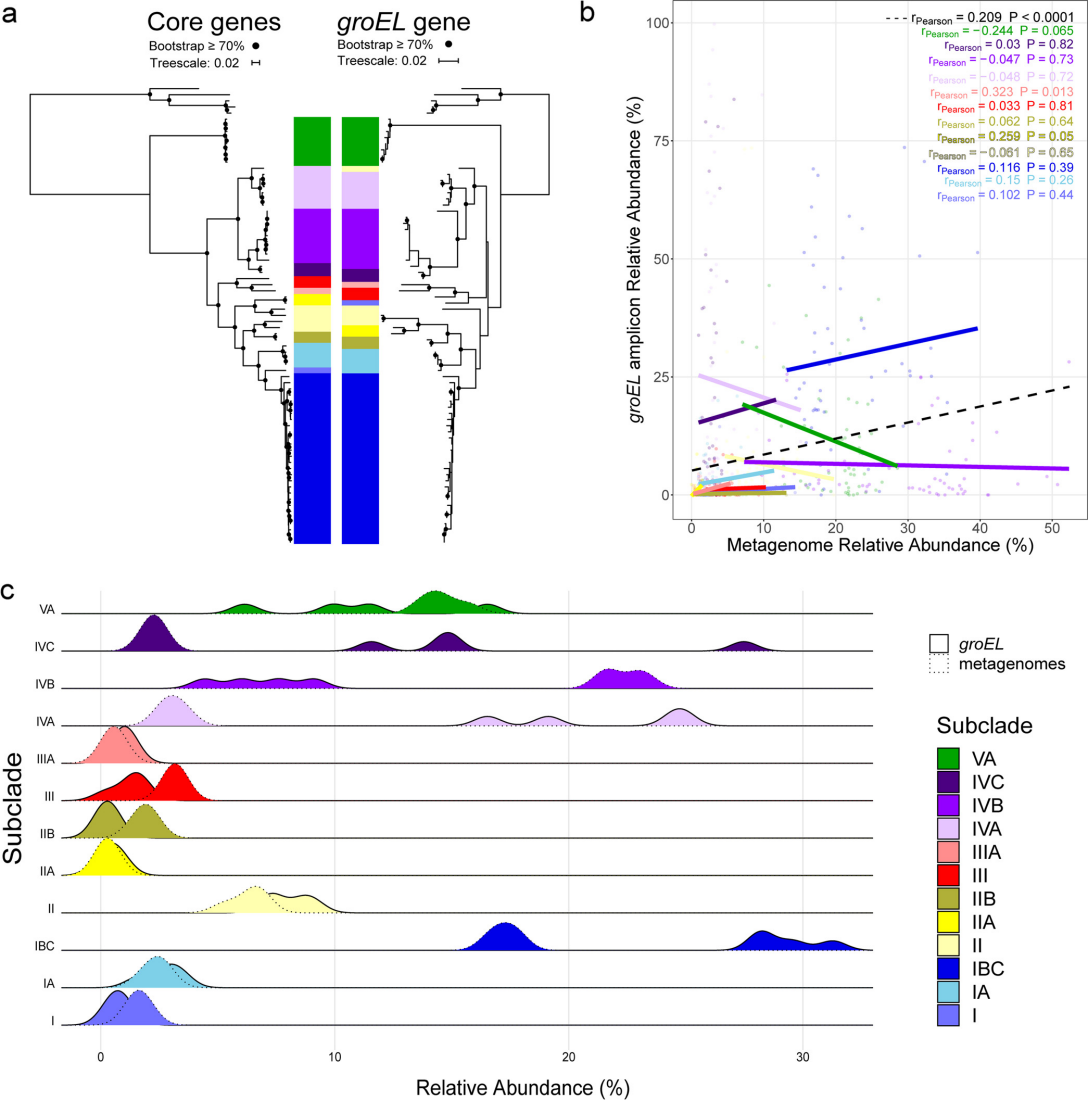
(a) Phylogenetic trees of the *Curtobacterium* genus created using 21 single-copy core genes (left [27]) and using only the *groEL* gene (right). The subclades are denoted by color, and at the top is the outgroup, *Frigoribacterium*, a closely related genus. Clades with bootstrap support of >70 are noted with a black circle at the node. (b) The relationship between the relative abundance of each subclade from the metagenomic analysis versus the relative abundance from the *groEL* amplicon analysis across the climate gradient. Each point represents a different sample that is colored by subclade. The overall correlation (dashed line) across all samples is plotted as reported in the top right of the figure. (c) Density plots of the relative abundance of *Curtobacterium* detected from the climate gradient samples. The *groEL* amplicon abundances are outlined by a solid line, and the metagenomic abundances, by a dotted line.

To evaluate the success of *groEL* as a marker for microdiversity within *Curtobacterium*, we used a two-pronged approach. First, we generated a phylogeny based on the targeted *groEL* region. The *groEL* phylogeny was highly congruent with a multilocus phylogeny based on 21 single-copy core genes (Fig. 1a; Mantel test, z = 0.749, *P* = 0.001). The genus can be divided into at least nine subclades, all of which could be distinguished by the targeted portion of the *groEL* gene. The overall topology of the two trees was also highly similar, but there were some differences in branching patterns (Fig. 1a). In particular, the clustering of the strains designated previously as clade II (light yellow in Fig. 1a) was uncertain, as was the placement of the two other strains (Wood-2 and Pine-20).

Our second test of the *groEL* marker was to compare the relative abundances of the subclades detected by classification of core genes in metagenomes versus *groEL* amplicons from the same samples. Approximately 52% of the *groEL* amplicon reads were classified as *Curtobacterium*. These sequences revealed a rough correspondence of the relative abundance of *Curtobacterium* subclades with that assessed by the metagenomic sequences in that the same subclades dominated the samples in both methods. For some subclades, such as II and VA, there was a close match in the relative abundance predicted by the two methods (Fig. 1c). Further, the three relatively low-abundance subclades (IIA, IIB, and IIIA) showed similarly low abundances by both methods; however, the relative abundances of closely related subclades IVA and IVB were reversed by the two methods (Fig. 1c). Lastly, while there was a weak overall correlation (*r* = 0.209, *P* < 0.0001), the relative abundances estimated by the two methods within subclades were not correlated (Pearson’s *R* range from −0.25 to 0.33), except for a significantly positive correlation within subclade IIA (*P* = 0.011; Fig. 1b). Thus, while the *groEL* approach provides a high-throughput method to track changes in relative *Curtobacterium* microdiversity across samples, it is likely not a good quantitative estimator of subclade abundance.

### Response to the global change treatments and ecosystem type.

**FIG 2:**
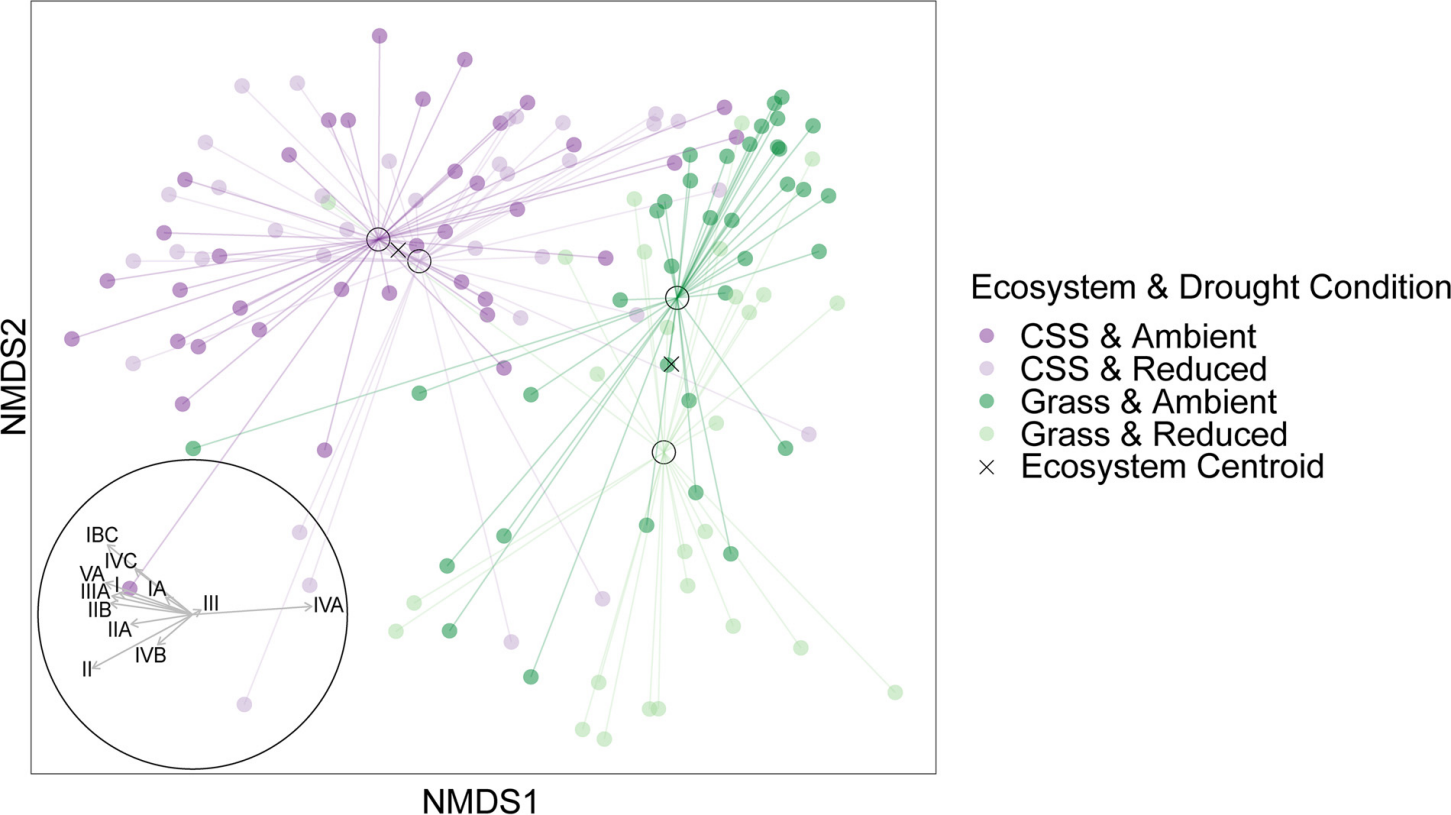
Nondimensional metric scaling (NMDS) plots of *Curtobacterium* microdiversity (ESVs of *groEL* amplicons) from the LRGCE samples using Bray-Curtis distance. Vectors on the bottom left represent the direction and strength of correlation with the *Curtobacterium* subclades. The centroids of each treatment combination are marked by a black circle, and the centroids of all samples in the two ecosystems are marked by an X, showing the greater effect of drought in the grassland.

**FIG 3:**
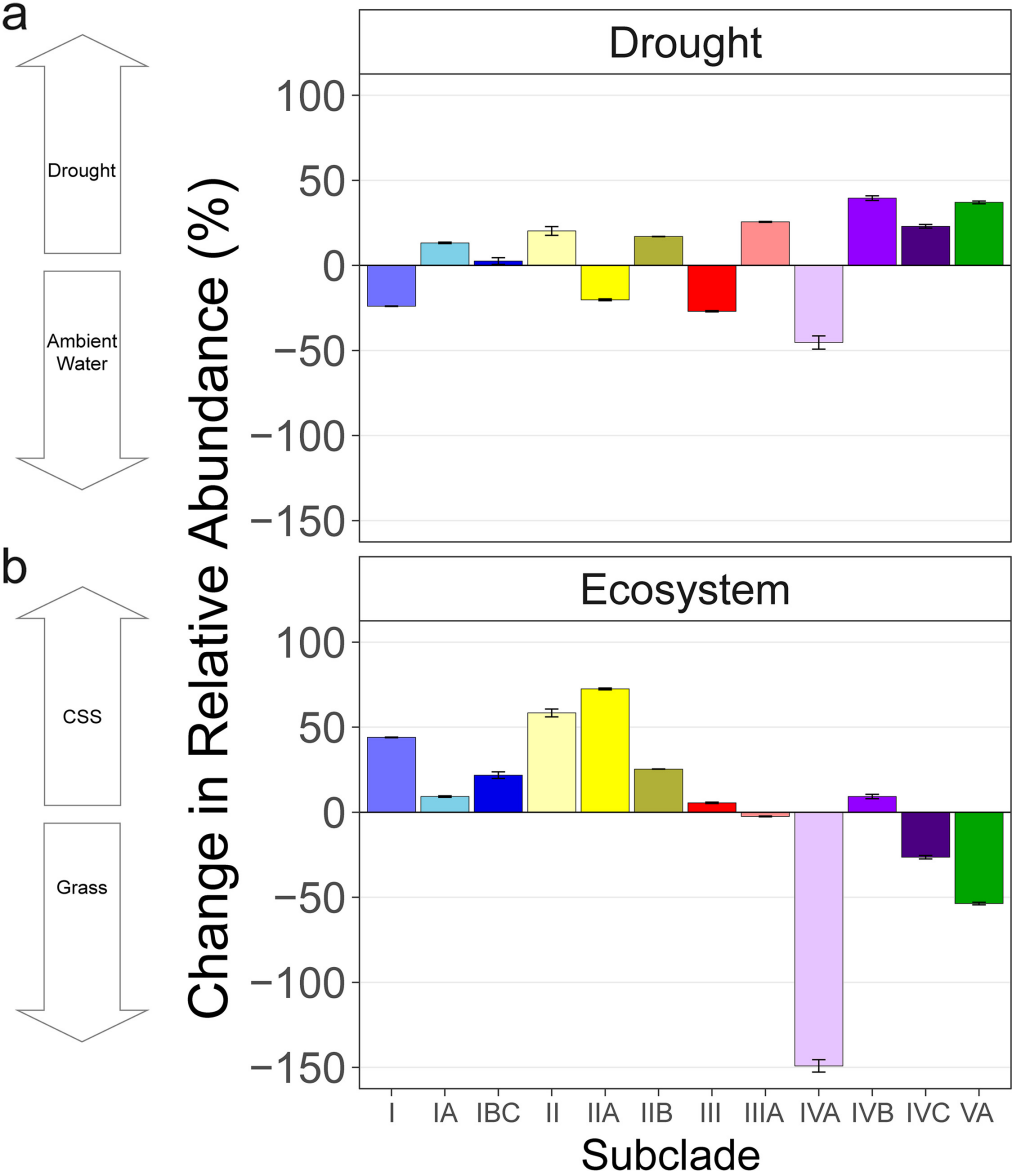
(a and b) Percent difference (mean ± standard error [SE]) in relative abundance of *Curtobacterium* subclades in (a) the drought versus ambient rainfall treatments and (b) the CSS versus grassland ecosystems (bottom panel). Subclades are colored as in Fig. 1. The percent change value for drought is calculated as the relative abundance under drought conditions minus the relative abundance under ambient conditions, divided by the relative abundance in drought. A positive value in the drought panel means higher relative abundance under drought conditions.

The composition of *Curtobacterium* microdiversity varied significantly between the drought and ambient treatments, across the two ecosystems, and over time. In particular, the composition of *groEL* ESVs within the genus responded to the drought treatment, explaining 2.8% of the variation (permutational multivariate analysis of variance [PERMANOVA], *P* = 0.001; Table 1; Fig. 2). As we know more about subclade distribution, we further analyzed at this level. Summarizing microdiversity by subclade, subclade IVA had a higher relative abundance under ambient conditions, whereas subclades IVB, IVC, and VA were more abundant in the drought treatment (Fig. 3a). In contrast to drought, added nitrogen did not alter *groEL* ESV composition.

**TABLE 1:** PERMANOVA results evaluating the contribution of date of sample collection, ecosystem (grassland or CSS), drought treatment, and nitrogen treatment to variation of *Curtobacterium* composition (as assayed by *groEL* ESVs)^*a*^^,^^*b*^

Source	*df*	SS	MS	Pseudo-F	P(Perm)	Estimated variation (%)
Ecosystem	1	2.6	2.7	7.4	**0.001**	10.4
Drought	1	0.9	0.9	2.6	**0.001**	2.8
Nitrogen	1	0.4	0.4	1.2	0.117	
Date	6	2.9	0.5	1.4	**0.004**	1.8
Ecosystem × drought	1	0.7	0.7	2.0	**0.002**	2.7
Ecosystem × nitrogen	1	0.4	0.4	1.2	0.124	
Ecosystem × date	6	2.3	0.3	1.1	0.112	
Drought × nitrogen	1	0.4	0.4	1.2	0.123	
Drought × date	6	2.1	0.3	0.9	0.474	
Nitrogen × date	6	1.8	0.3	0.9	0.974	
Residuals	104	37.1	0.3			82.3
Total	134	52.9				

a *df*, degrees of freedom; SS, sums of squares; MS, mean squares.

b Variation estimates are reported for statistically significant variables (indicated in bold).

Ecosystem (grassland or CSS) contributed the largest amount of variation in *Curtobacterium* microdiversity, accounting for 10.4% of the total variation (*P* = 0.001), and the temporal sampling accounted for an additional 1.8% (*P* = 0.004). These differences across ecosystems were largely driven by particular subclade preferences; for instance, subclade IVA was 150% more abundant in the grassland, while clade II and associated subclades (IIA and IIB) preferred the CSS (Fig. 3b). Ecosystem and drought also interacted to explain 2.7% of the variation in *Curtobacterium* ESV composition (PERMANOVA, *P* = 0.002). This interaction can be visualized in an ordination plot by the pronounced effect from the drought treatment on the grass samples but had little discernible effect on the CSS samples (Fig. 2, ecosystem centroids).

### Genomic and metagenomic CAZyme variation.

**FIG 4:**
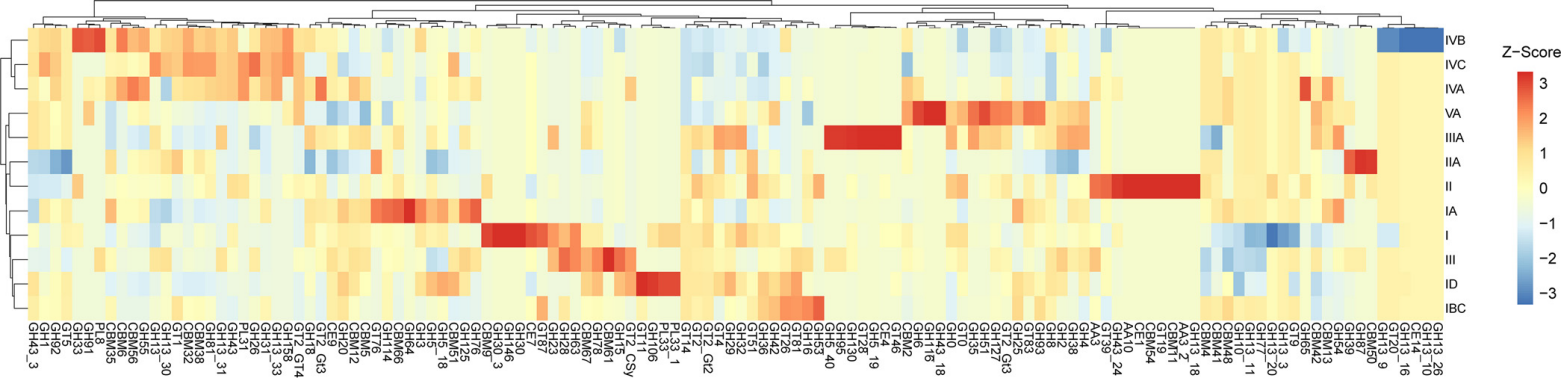
Heatmap of normalized mean CAZyme abundances of *Curtobacterium* clades. Data were normalized to a mean of 0 and a standard deviation of 1. CAZymes and clades were grouped by hierarchical clustering using Ward’s method and Euclidean distances.

While the global change treatments influenced subclade distributions, the majority of variation was explained by the two ecosystems, likely reflecting differences in litter chemistry. Therefore, we targeted functional traits directly associated with carbohydrate degradation by investigating the CAZyme genomic content across subclades. The number of CAZymes across all *Curtobacterium* genomes (*n* = 143) ranged from 61 to 84 genes, with a median of 70 CAZymes per genome. Across subclades, there was significant variation in the total number of CAZymes (see Fig. S1 in the supplemental material; Kruskal-Wallis *P* < 0.001), as well as significant compositional variation (analysis of similarity [ANOSIM], *R* = 0.685, *P* = 0.001). Major CAZymes driving the differences between some of the subclades include GH158, found in subclades IVA and IVB and thought to hydrolyze fungal laminarin, and GH116, found in subclade VA and thought to hydrolyze glucose and xylan (Fig. 4).

**FIG 5:**
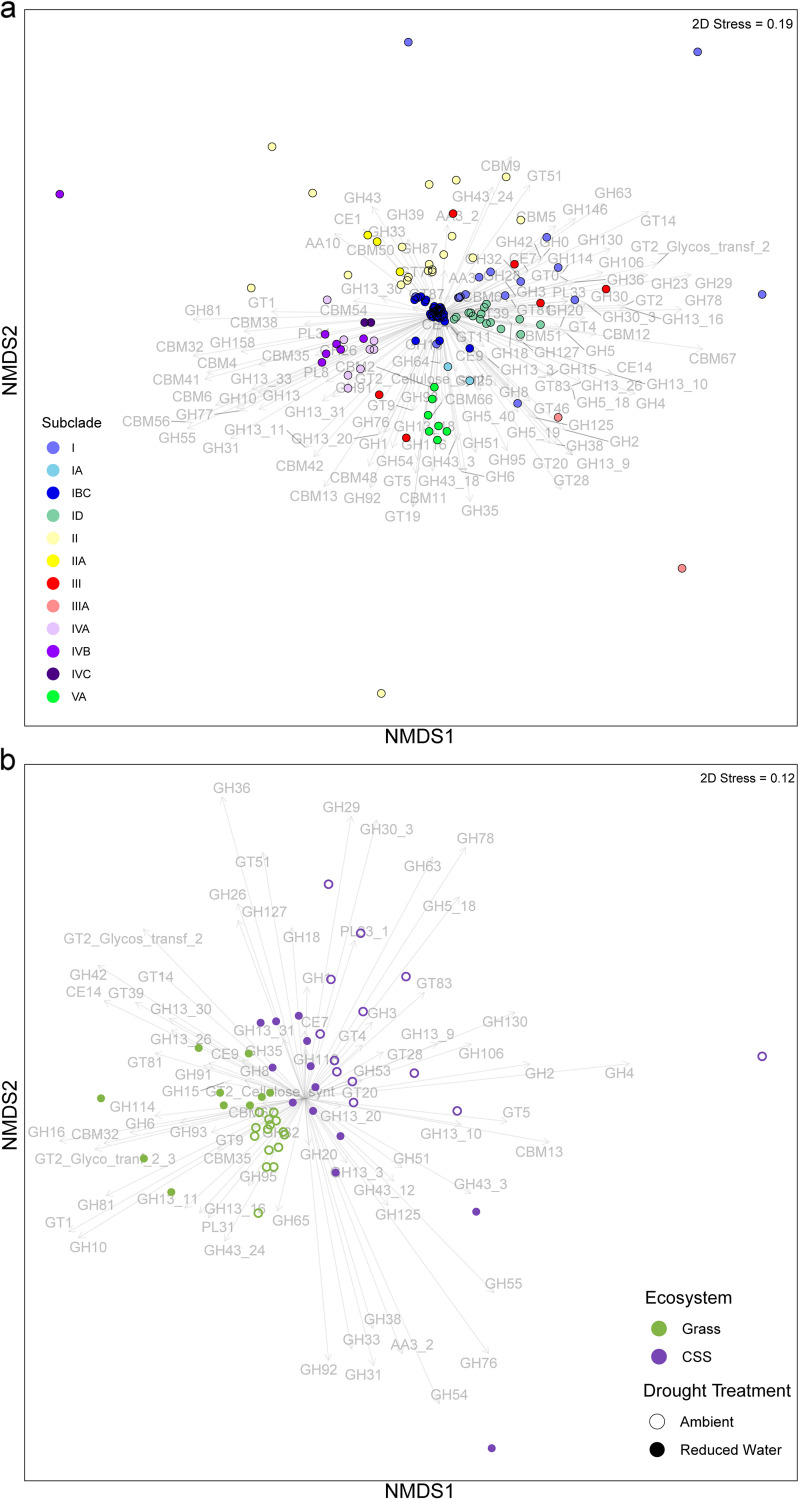
(a) Nondimensional metric scaling (NMDS) plot showing the differences in genomic CAZyme content among *Curtobacterium* strains, colored by subclade. Overlaid are vectors whose direction and magnitude reflect the correlation of the CAZymes to the NMDS axes. (b) NMDS plot of *Curtobacterium* CAZyme content from metagenomic samples in the experimental plots at the LRGCE. Vectors represent the correlation of CAZymes to the NMDS axes.

Lastly, we investigated CAZyme variation in *Curtobacterium* within the LRGCE treatments in metagenomic libraries. There was no significant difference in the overall CAZyme count between ecosystem or drought treatment. However, as with *groEL* ESV composition, the composition of *Curtobacterium* CAZymes varied significantly between the drought and ambient treatments, across the two ecosystems, and over time. The largest effect was from ecosystem, also consistent with *groEL* composition, explaining 18.6% of the total variation (Table 2; PERMANOVA *P* = 0.001). CAZyme composition was highly distinct between ecosystems (Fig. 5; *P* = 0.001), and this strong division was driven by small but highly consistent differences in the relative abundances of many CAZymes (Fig. S2). There was also variation in dispersion, which can contribute to significant compositional effects in a PERMANOVA test (32), with significantly less dispersion in the grass ambient samples than the CSS ambient, grass drought, or CSS reduced samples (PERMDISP; *P* = 0.001; Fig. 5b).

**TABLE 2:** PERMANOVA results evaluating the contribution of ecosystem, drought treatment, and date of sample collection on Curtobacterium CAZyme composition from metagenomic samples^*a*^^,^^*b*^

Source	*df*	SS	MS	Pseudo-F	P(Perm)	Estimated variation (%)
Ecosystem	1	1,057.8	1,057.8	26.2	**0.001**	18.6
Drought	1	300.6	300.5	7.4	**0.001**	4.8
Date	3	614.0	204.6	5.1	**0.001**	5.9
Ecosystem × drought	1	438.4	438.3	10.9	**0.001**	14.6
Ecosystem × date	3	447.9	149.3	3.6	**0.001**	7.8
Drought × date	3	298.3	99.4	2.5	**0.001**	4.2
Ecosystem × drought × date	3	261.6	87.2	2.2	**0.001**	6.7
Residuals	89	3,592.4	40.4			36.3
Total	104	7,042.4				

a *df*, degrees of freedom; SS, sums of squares; MS, mean squares.

b Variation estimates are reported for statistically significant variables (indicated in bold).

## DISCUSSION

Closely related bacteria co-occur in the same habitat (30, 33), and such microdiversity suggests that coexistence may be promoted by fine-scale variation in traits (12, 14, 34). Overall, our results indicate that aggregating genetic diversity at the 16S level at least partially masks disparate responses to global change at finer scales of genetic variation. Previous work found that the relative abundance of *Curtobacterium* increased under drought conditions (6, 28). We find further variability in the response to drought within the genus, supporting our first hypothesis that microdiversity responds disparately to the global change simulations. However, in contrast to our expectation that the response to added nitrogen would be stronger than that to drought, *Curtobacterium* microdiversity did not respond to nitrogen addition. Nitrogen addition often affects bacterial community composition in other soil systems at the 16S level (35–37), including in these same samples from the LRGCE system (28). This result suggests that the response to nitrogen addition may be more conserved than that of drought within *Curtobacterium*, in contrast to a meta-analysis that found that the phylogenetic depth of response across all bacteria was similar for various simulated global changes (20).

A main reason that we selected the *groEL* gene as a taxonomic marker is because its phylogeny and (presumably) evolutionary history are well matched to that of the 16S region and other markers. This suggests that it evolves relatively slowly compared to most parts of the genome and is not subject to high levels of horizontal gene transfer (HGT). Indeed, we found that variation in the *groEL* sequence reflected the phylogenetic distinctiveness of *Curtobacterium* subclades, indicating that shifts in *groEL* diversity will generally reflect relative shifts in *Curtobacterium* microdiversity. A further benefit of using a protein-coding gene over the 16S region to study microdiversity is that much of the genetic diversity will encompass synonymous mutations (in many first and third codon positions). In contrast, the 16S region is famously slow in its evolution, presumably because much of it is under stabilizing selection (38). The high number of synonymous mutations thus allows protein-coding regions such as *groEL* to resolve finer-scale differences in diversity than even variable regions of the 16S region can. Of course, selection for particular *groEL* genes in our treatments is possible; the *groEL* gene codes for a stress response chaperonin, and mutations therein may confer tolerance to different stress conditions (39, 40). However, the vast majority of genetic variability observed in *groEL* diversity is likely neutral with respect to our treatments and, because HGT of the gene is rare, linked to other regions in the genome that are under selection. Thus, we conclude that targeting *groEL* to assay shifts in fine-scale genetic composition is a useful approach for microdiversity exploration (16, 41–43).

Our second hypothesis, that the response of microdiversity would depend on other traits, was supported by the large ecosystem effect, which mirrors the disparate chemical signatures of the litter substrates between the grassland and CSS ecosystems (31). This is further supported by the interactive effect between treatment and ecosystem, which is larger even than the main drought effect. The microdiversity response may be influenced by selection on other shallowly conserved traits such as CAZymes that might be easily shared via horizontal gene transfer among closely related strains within a genus. For example, subclade IVA encodes a high proportion of chitinases and laminarinases compared to other clades, and the target substrate for these enzymes is likely fungal cell walls, as their major component is β-1,3/1,6-glucan (44). Thus, specialization on fungal necromass versus other substrates may allow for resource partitioning to promote the coexistence of microdiversity. We suspect that these functional traits contribute to environmental distributions; in this case, subclade IVA was more abundant in the grassland, where the fungal to bacterial ratio is significantly greater than in the CSS (31). Supporting this idea, we further found that the composition of *Curtobacterium-*specific CAZymes responded strongly to drought as well as the ecosystem.

That said, there was not an obvious relationship between shifts in the abundance of particular subclades by treatment or ecosystem and the relative abundance of *Curtobacterium* CAZymes in metagenomic samples, even though the subclades differ distinctly in their genomic CAZyme content. This lack of correspondence between our results could suggest that we have not adequately isolated the genomic diversity of *Curtobacterium* and/or that *groEL* amplification is not a quantitative marker of the subclades. Indeed, *groEL* amplicon abundances correlated only weakly with *Curtobacterium* subclade abundances from the metagenomes. However, assigning metagenomic sequences at a fine phylogenetic scale is also challenging, so these may not reflect true subclade abundances.

While we did not observe a clear correlation between taxonomy and CAZyme composition, we did detect a strong response across ecosystems. Notably, the response (assessed by *groEL*) of *Curtobacterium* microdiversity to drought in the grassland was much larger than that in the CSS. Bacteria on the more recalcitrant CSS litter may be required to invest most of their cellular energy in degrading the leaves, perhaps revealing a trade-off in microbial life history strategies (45–48). However, a similar interactive pattern was not reflected in the composition of *Curtobacterium* CAZymes, as these genes responded distinctly in both ecosystems, albeit in a different way. This discrepancy may indicate that the sensitivity of *Curtobacterium* microdiversity to drought depends not only on CAZymes but on additional traits not considered here.

In conclusion, the responses assessed at the 16S level reflect the summation of divergent responses among finer-scale taxa. However, it is important to distinguish between the type of information that the 16S and *groEL* analyses provide. Because the *groEL* primers target only *Curtobacterium*, the analysis does not provide information about how *Curtobacterium* changes relative to all other bacterial taxa. Thus, the 16S analysis still provides unique and useful information about *Curtobacterium*, even when only one 16S OTU is effectively present. Separately, the *groEL* analysis demonstrates that it would be incorrect to extrapolate from the 16S data that all *Curtobacterium* respond relatively positively to drought. In contrast, the 16S responses to nitrogen addition seem to apply throughout the genus at our field site. The varied responses to simulated drought within a genus of soil bacteria demonstrate the potential importance of finely conserved traits—traits that are otherwise aggregated by the broader taxonomic shifts observed in most microbial global change studies. Of course, it is not feasible to assess every bacterial genus’ response to global change at a finer and finer scale of diversity. However, future investigations from closely studied models such as *Curtobacterium* could identify traits involved in responses to environmental change, and the genetic resolution is needed to assess them. Such studies could thereby improve predictions about global change responses of the wider microbial community and consequences for soil ecosystems.

## MATERIALS AND METHODS

### Field sites.

The Loma Ridge Global Change Experiment (LRGCE) was established in Irvine, CA, USA (33°44′N, 117°42′W, 365 m elevation) in 2007. Precipitation and nitrogen treatments are applied in two adjacent ecosystems, deciduous shrubland (coastal sage scrub, CSS) and annual grassland (49). The grassland plots are dominated by *Avena*, *Bromus*, and *Lolium*, and the CSS plots are dominated by *Artemesia* and *Salvia* (50). The site contains 48 experimental plots, 6.1 by 12.2 m in the grassland, and 18.3 by 12.2 m in the CSS. For this study, we sampled from four replicate plots from four treatments (drought, added nitrogen, drought and added nitrogen, and control) in both the grassland and CSS habitats. Since 2007, the drought treatment (∼50% reduction of annual ambient rainfall) was achieved by intercepting rainfall with a large polyethylene sheet during approximately half of the rainstorms. Nitrogen addition was achieved by adding soluble CaNO_3_ (60 kg N^−1 ^ha year^−1^ added) (49).

### *groEL* sequencing and analysis.

To identify a marker gene that enabled the characterization of the microdiversity within *Curtobacterium*, we initially identified 12 orthologous proteins that could distinguish between *Curtobacterium* subclades. Based on the feasibility to amplify the flanking regions of these candidate genes, we selected the *groEL* gene that encodes part of the *groEL-groES* chaperonin complex (51). Notably, the *groEL* gene has been used as an alternative to small subunit rRNA genes in other taxa (52). The final primer design targeted a 20-bp region at each end—(Curto-groEL-72F) CGCCGTGAAGGTGACGCTCG and (Curto-groEL-450R) GCCGAGATCGACGCSGTSGC. To test the specificity of the primers, we confirmed amplification of the correct size band from six *Curtobacterium* isolates spread across the phylogeny. We also tested that we did not amplify the region from leaf litter isolates of the closely related genus *Frigoribacterium* or other distantly related taxa such Pseudomonas (two genera that are commonly found in the LRGCE leaf litter communities).

*Curtobacterium* microdiversity was characterized on 219 leaf litter samples collected from 3 areas of each treatment plot at 7 time points (once per season) over a year and a half—22 August 2016, 12 December 2016, 30 March 2017, 28 June 2017, 13 September 2017, 13 December 2017, and 29 March 2018. In the lab, the leaf litter was ground using a coffee grinder, and DNA was extracted from 0.05 g using the ZymoBIOMICS DNA miniprep kit (Zymo Research; Irvine, CA). We added 3 μL of this DNA to a 25-μL PCR cocktail containing 12.5 μL AccuStart II PCR ToughMix 2× reagent (Quantabio; Beverly, MA) and 1 μL each of the *groEL* primers, and the remainder was made up with nuclease-free water. PCR conditions were as follows: 94°C for 5 min, followed by 27 cycles of 94°C for 45 s, 68°C for 45 s, and 72°C for 45 s, with a final extension of 72°C for 10 min. The amplicons were purified using a 1:1 ratio of AMPure beads and eluted into 20 μL nuclease-free water. Then, 1 μL each of unique i5 and i7 Nextera (Illumina, Inc., San Diego, CA) indices was added to each sample, followed by a second PCR under the following conditions: 94°C for 3 min, followed by 8 cycles of 94°C for 30 s, 55°C for 30 s, and 72°C for 30 s, with a final extension of 72°C for 10 min. Amplified DNA was quantified using a Qubit instrument (BioTek, Winooski, VT) and assessed for quality using a Nanodrop instrument (Thermo Fisher, Waltham, MA). Samples were prepared according to the Nextera XT library preparation kit (Illumina, Inc.). The samples were then pooled equimolarly and cleaned up using a 1:1 ratio of AMPure beads, eluted into 100 μL, and sequenced on an Illumina MiSeq instrument with 300-bp paired-end reads, though the reverse reads were not used due to low quality.

We used a QIIME 2 v2018.11 (53) pipeline to denoise and trim unprocessed FASTQ sequence files with DADA2 (54). To classify *groEL* amplicons at the subclade level, we first clustered sequences to exact sequence variants (ESVs). To validate that ESVs belonged to *Curtobacterium* and were not erroneously assigned, we screened the amplicon sequences against a custom database (since we used a non-16S marker gene with custom primers) containing 70 *groEL* sequences extracted from publicly available genomes of *Curtobacterium* (27), as well as 10 other major taxa commonly found in leaf litter communities to serve as outgroups. Using a trained classifier from the feature-classifier QIIME plugin (55), we used phylogenetic inference of the *groEL* gene to place ESVs (56) into a maximum likelihood phylogeny generated from the 70 reference sequences built with RAxML v8.2.12 (57) under the GTRGAMMA model with 100 replicates.

To validate the *groEL* amplicon approach, we compared our results to previously published shotgun metagenomic data. While the *groEL* sequences provide a much finer resolution of genetic diversity, we first amplified the *groEL* region from 96 samples from a regional climate gradient in southern California that included the LRGCE. We compared the *groEL* diversity with the shotgun metagenomic results at the clade level (five clades originally reported in [27]). In an earlier study, *Curtobacterium* abundances from the metagenomic libraries were calculated with a multilocus sequence assignment using 21 single-copy genes specific to *Curtobacterium* (27). Separately, we placed the amplified *groEL* sequences on a phylogenetic tree of *Curtobacterium groEL* sequences using the SEPP plugin in QIIME2 (56). Approximately 52% of the ∼10,000 ESVs were assigned to *Curtobacterium*. Of these, 88% were assigned to the species level, with the remaining 12% only placing to genus level, indicating that most of the genetic diversity was captured by the *Curtobacterium* genomes in the phylogenetic tree. The samples had a mean of 51% *Curtobacterium* hits (±25.3% standard deviation), and the non-*Curtobacterium* reads were removed from further analysis. For both the metagenomes and our amplicon method, the relative abundance of each subclade was calculated by dividing the number of reads assigned to a subclade by the total number of assigned reads in that sample.

### *Curtobacterium* CAZyme composition.

To assess CAZyme diversity within *Curtobacterium* genomes, we downloaded all available (*n* = 143) *Curtobacterium* genomes from NCBI and analyzed their CAZyme profile using the program dbCAN2 (58) with the HMMdb v9 database released on 4 August 2020 and the latest CAZyDB, released on 30 July 2020 (59). dbCAN2 uses a 3-fold approach to identify CAZymes, and we only retained CAZymes that were detected by all three approaches. CAZyme abundances were normalized across genomes by calculating a Z-score with a mean of 0 and a standard deviation of 1, after which we performed an nondimensional metric scaling (NMDS) analysis and plotted the correlation vectors.

From the detected *Curtobacterium* CAZymes in the genomes, we built a custom database to identify the abundance of *Curtobacterium* CAZymes in metagenomic sequences from the LRGCE treatments. The sequences were checked for quality using FastQC v0.11.9 (60), after which they were quality filtered and trimmed using Trimmomatic v0.39 (61). We then used DIAMOND BLASTX (62) on the forward reads, taking into account all six putative open reading frames, for comparison to the reference database. To assess the validity of hits, we used CAZymes from the closely related sister genus *Frigoribacterium* to benchmark our thresholds and set positive hits at an E value of 1e^−20^ and an identity of >98%. Finally, we performed a reciprocal BLAST search of the putative hits against the full NCBI nonredundant protein database to confirm the reads identified as *Curtobacterium*.

### Statistical analyses.

To assess the contribution of the global change treatments on *Curtobacterium* composition, we performed a permutational multivariate analysis of variance using PRIMER6 with PERMANOVA+ (Primer‐E Ltd., Ivybridge, UK) (63). The *groEL* PERMANOVA model included four factors, ecosystem, date of sample collection, precipitation treatment, and nitrogen treatment, as fixed effects. Compositional similarity between samples was calculated using the Bray-Curtis dissimilarity metric after rarefying to a sample depth of 5,000 sequences with 100 repetitions. We ran a type III partial sum of squares PERMANOVA for 999 permutations of residuals. Variation explained was calculated by summing the estimated components of variation for the statistically significant terms and the residuals and dividing each by this total (64). We followed the same process for the metagenomic PERMANOVA model, although there was no nitrogen addition treatment. The *Curtobacterium* genomic CAZyme content data were normalized by centering to a mean of 0 and a standard deviation of 1 using the scale function in R. ANOSIM and SIMPER analyses to assess differences in subclade CAZyme composition used Bray-Curtis dissimilarities and were performed with the vegan package in R (65).

### Data availability.

The raw amplicon and metagenomic reads can be accessed under the NCBI BioProject accession number PRJNA781975. The BioSample accession numbers are SAMN23310455 for the *groEL* amplicons and SAMN23390019 for the metagenomic reads.
