# Supplementary material for: Differential Response of Bacterial Microdiversity to Simulated Global Change
Source: Appl Environ Microbiol. 2022 Mar 22;88(6):e02429-21. doi: 10.1128/aem.02429-21 (PMC8939344; doi:10.1128/aem.02429-21)
Supplement: Supplemental file 1 — Fig. S1 and S2. Download aem.02429-21-s0001.pdf, PDF file, 2.2 MB [file aem.02429-21-s0001.pdf]

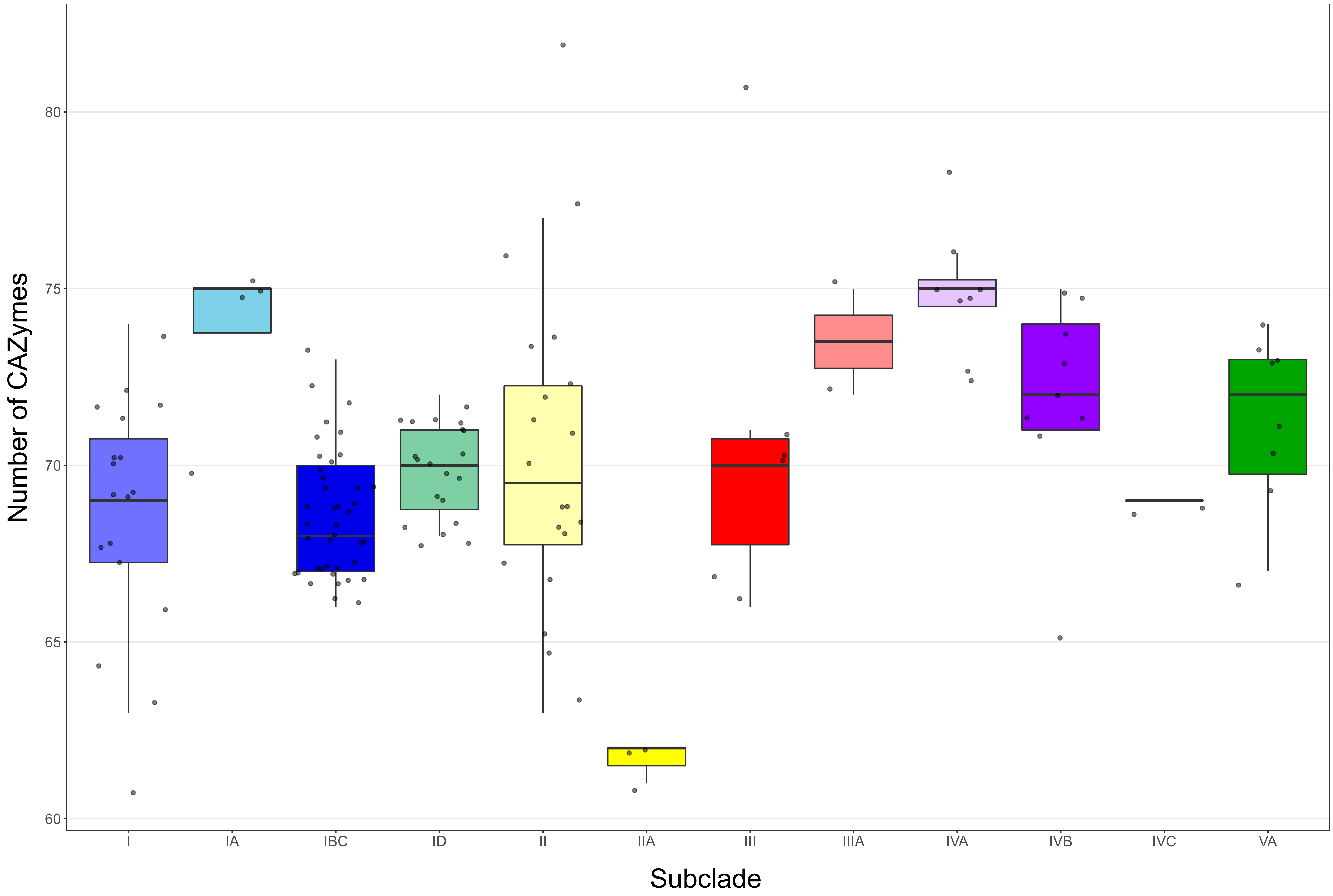

Figure S1. Curtobacterium genome CAZyme counts. Each point represents the number of CAZyme genes in a genome, colored by subclade.

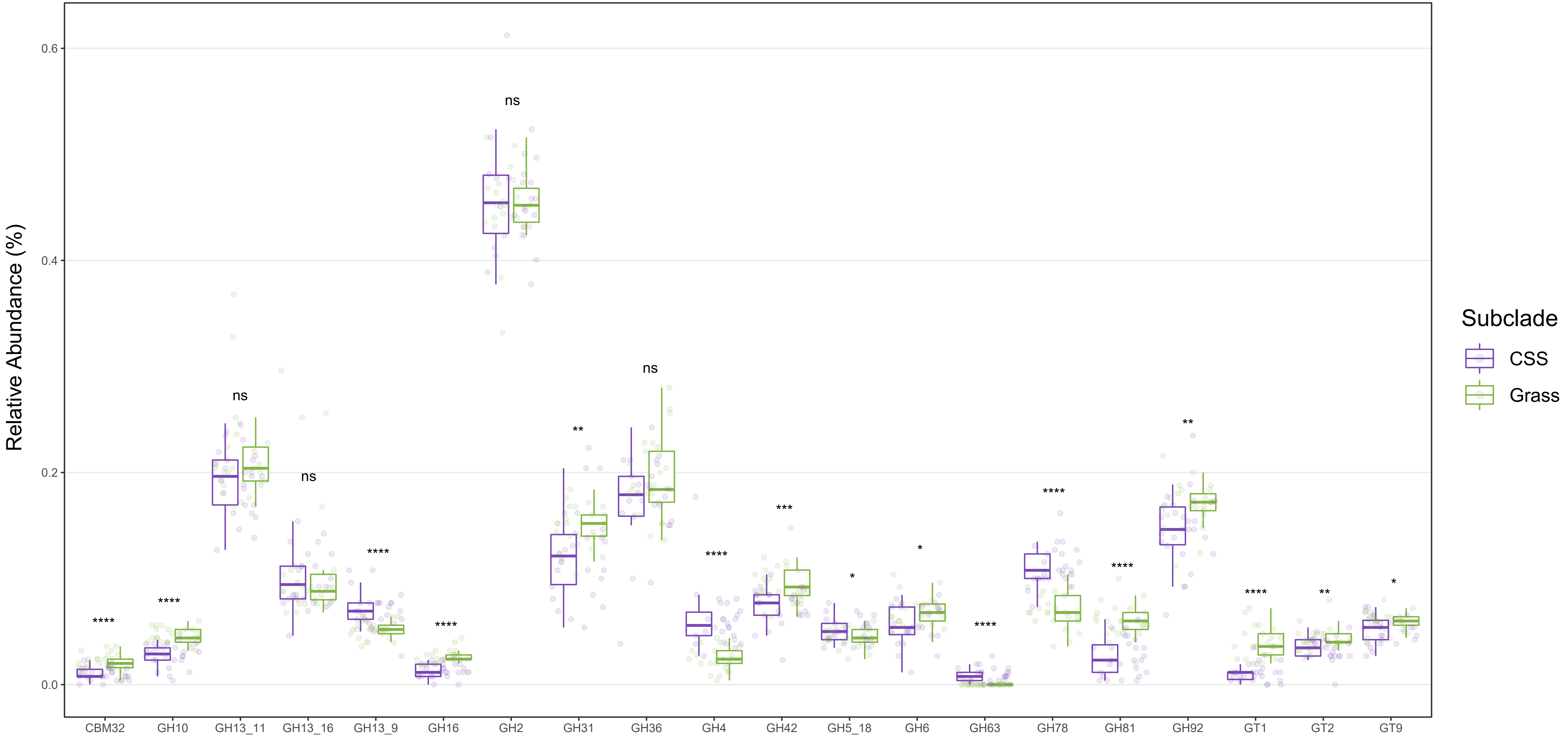

Figure S2. Relative abundance of CAZymes across ecosystem. Pairwise Wilcoxon test results shown.
